# Editorial: Advanced Biosensing Technologies in Medical Applications

**DOI:** 10.3390/bios13010011

**Published:** 2022-12-22

**Authors:** Han-Sheng Chuang

**Affiliations:** 1 Department of Biomedical Engineering, National Cheng Kung University, Tainan 701, Taiwan; oswaldchuang@mail.ncku.edu.tw; 2 Medical Device Innovation Center, National Cheng Kung University, Tainan 701, Taiwan

The unprecedented pandemic over the past three years has accelerated the developments of many cutting-edge techniques to address the challenges raised in new medical frontiers. This crisis, though may be devastating on one hand, provides us with another chance to look into our deficiency on the other hand. Among three common methods in epidemic prevention, i.e., vaccination, diagnosis, and medication, diagnosis appears to be the cost-effective and simple measure to prevent diseases in the early stage. Considering numerous breakthroughs have been enabled in recent years by new materials, such as graphene, carbon nanotubes, molecular beacons, quantum dots, etc., and new micro/nano-fabrications, such as ultra-fast laser machining, atomic layer etching, electron-beam lithography, etc., it is natural to ask a question in regard to how these innovations can be contributed to the biosensing technology, especially in medical applications. Followed by this emerging tendency, this special issue was organized to promote the significance of advanced biosensing technologies and hopefully inspire more potential solutions to many other unmet medical needs. Overall, a total of nine papers were published under the special issue, including six regular research papers and three review articles. The regular research papers introduce optical sensing (surface plasmon resonance, interferometry, and spectrometer) and mechanical sensing (diffusometry and size fractionation) in a variety of medical applications (nasopharyngeal carcinoma, human immunodeficiency virus (HIV), diabetic retinopathy, lung cancer, and bacterial infections). The review articles are involved with three medical topics in need of different levels of biosensing that cover diabetes management, malaria detection, and chronic non-communicable diseases. The research highlights of the collected papers are briefly summarized in the following to facilitate readability.

Among the research papers, two of them are serial publications associated with diffusometric biosensors [1,2]. Yang et al. [2] first reported a novel approach based on Brownian motion to enable rapid antimicrobial susceptibility. Four motile and non-motile bacteria (*Escherichia coli*, *Staphylococcus aureus*, *Pseudomonas aeruginosa*, and *Klebsiella pneumoniae*) mixed with six antibiotics (ampicillin, cefazolin, Baktar, ciprofloxacin, daptomycin, and gentamicin) that cover five antiseptic mechanisms were investigated. Their findings showed that the temporal diffusivities of both motile and non-motile bacteria stopped declining after a certain time point when the microbes were inhibited by the antibiotics. In a single-blinded clinical test, they concluded that the sensitivity, specificity, and accuracy of the system could reach up to 92.9%, 91.4%, and 91.8%, respectively. In a later study, Chen et al. [1] further advanced their translation diffusometry with Janus particles. In combination with Stoke–Einstein and Stoke–Einstein–Debye relations, they successfully removed the background viscosity and temperature, leaving only the particle diameter as an output. The slightly modified algorithm imparted the new diffusometry, 96-fold and 15-fold more stable than the old system regarding viscosity and temperature changes, respectively. As a result, the limit of detection (LoD) was brought down to nearly 0.45 pg/mL.

In another study, Guzman et al. [3] attempted to measure the tear-based biomarker protein, lipocalin 1 (LCN-1), for simple diabetic retinopathy screening. The device was made up of a capillary filled with a porous hydrogel (pore size = 45 μm) to sieve capture particles (d_p_ = 40 μm) as well as gold nanoparticles (AuNPs). In the presence of LCN-1, each sandwiched immunocomplexed particle would be covered with AuNPs. Therefore, the immunocomplexed particles accumulated in the entrance of the hydrogel exhibited color, indicating the presence of target proteins. The optimal LoD was claimed to reach 1 ng/mL. In an evaluation of clinical samples, significant difference in the LCN-1 levels between the healthy subjects and patients with PDR/NPDR implied the potential possibility to identifying DR with the proposed diagnostic tool. 

Next, Chupradit et al. [4] used biolayer Interferometry (BLI) as a label-free technology for determining kinetic biomolecular interactions. They determined the kinetic binding of a zinc finger scaffold, 2LTRZFP, which formerly constructed the interfering effect on ds2LTR HIV-1 integration process using BLI. The binding affinity of 2LTRZFP against ds2LTR target analyzed by BLI was 40 nM. The results indicated that 2LTRZFP-GFP binds specifically to ds2LTR target and does not display off-target effects to human chromosome. They proved that BLI is likely a reliable technique for determining the protein and nucleic interaction.

In another case of diagnosing of nasopharyngeal carcinoma (NPC), Lo et al. [5] once employed a micro/nanostructure co-hot embossing technique to fabricate gold-capped nanostructures as localized surface plasmon resonance sensors in a microfluidic channel. This point-of-care device allows the NPC patients to reliably track their disease with a biomarker, LMP-1, even when they are not in the hospital. In the calibration, different concentrations of LMP-1 samples were continuously flown into the microfluidic channel at a flow rate of 10 µL/h for 10 min to dynamically conjugate the LMP-1 onto the anti-LMP-1 in the sensing region. This paper demonstrated that the device was capable of reaching a lower concentration of 30 ng/mL.

Besides NPC, lung cancer receives attention from some research groups as well. Mitrayana et al. [6] focused on developing a CO_2_ laser photoacoustic spectrometer for measuring acetone in the breath from lung cancer patients. Notably, breath acetone has been widely considered as a potential biomarker of some diseases. In addition, two other volatile organic compounds (VOCs), ethylene and ammonia, were investigated for comparison in this study. Eventually, the authors stated that their CO_2_ laser PAS measured the LoDs for the ethylene, acetone, and ammonia VOCs down to 6 ppbv, 11 ppbv, and 31 ppbv, respectively. Their finding indicated that the concentration of breath acetone detected in the lung cancer group appeared to be higher than those in the other lung disease group and the healthy group.

Other than the regular research papers, three review articles are also collected in this special issue to provide insightful discussions of advanced biosensors in some medical fields. Rsscalli et al. [7] discussed an issue of analytical challenges in diabetes management. Instead of the glycated hemoglobin (HbA1c), which is currently the “gold standard” for diabetes screening and monitoring in clinics, they argued that glycated albumin (GA) has more potential to be a control biomarker due to its shorter lifespan and wider reliability. To this end, this review describes the most up-to-date advances in the field of glycemic control biomarkers, exploring in particular the GA with a special focus on the recent experimental analysis techniques, using enzymatic and affinity methods. In addition, this review also collects analysis steps and fundamental reading technologies that can be integrated into a processing pipeline, hoping to pave a way for future point-of-care testing (POCT). In another review, Baptista et al. [8] focused on microchip-enabled malaria detection to provide prompt, accurate and sensitive malaria diagnosis, which remains an unmet need to date. Hemozoin is accepted here as a unique malaria biomarker in the current practice because hemozoin crystals are produced as the parasites invade the red blood cells and their content relates to disease progression. Electrochemical and optical biosensors plus lab-on-chip (LoC) microdevices in the end were considerably discussed in the article. The LoC microdevices, despite being under intensive development and not yet ready for clinical use, indeed show promising prospects in speed, point-of-care, and detection limit. The authors believed the LoC biosensors based on hemozoin detection should play a crucial role in the future malaria control and elimination. In the last collection, a systematic review for biomolecules and electrochemical tools in chronic non-communicable disease (NCD) surveillance was reported by Morais and colleagues [9]. Their study was inspired by a less noticeable but significantly increasing fact about NCDs that has been observed in individuals of all ages, making it one of the main leading causes of death worldwide. After screening a total of 47,706 articles from the database, only 178 articles fell within the conditions and were reviewed. Electrochemistry was selected here for the surveillance of common NCDs considering it as the most suitable facility for an early and accurate vigilance of health disorders. In the end, the authors recommended development of new non-invasive and reliable electrochemical methodologies for the detection and quantification of small variations in the concentration of biomarkers that allow the early diagnosis and adequate monitoring of NCDs.
